# Supreme™ laryngeal mask airway use in general Anesthesia for category 2 and 3 Cesarean delivery: a prospective cohort study

**DOI:** 10.1186/s12871-017-0460-x

**Published:** 2017-12-19

**Authors:** Shi Yang Li, Wei Yu Yao, Yong Jin Yuan, Wen Shu Tay, Nian-Lin Reena Han, Rehena Sultana, Pryseley N. Assam, Alex Tiong-Heng Sia, Ban Leong Sng

**Affiliations:** 1 Quanzhou Macare Women’s Hospital, Department of Anesthesiology and Perioperative Medicine, Quanzhou, 362000 China; 2 Department of Anesthesiology, Qinghai University Affiliated Hospital, Xining, Qinghai China; 3 Department of Women’s Anaesthesia, KK Women’s and Children’s Hospital, 100 Bukit Timah Road, Singapore, 229899 Singapore; 4 Division of Clinical Support Services, KK Women’s and Children’s Hospital, 100 Bukit Timah Road, Singapore, Singapore; 5 Centre for Quantitative Medicine, Duke-NUS Medical School, Singapore, Singapore; 6 Singapore Clinical Research Institute, Singapore, Singapore; 7 Duke-NUS Medical School, 8 College Road, Singapore, 169857 Singapore

**Keywords:** Supreme™ laryngeal mask, Airway management, General anesthesia, Emergency Cesarean section

## Abstract

**Background:**

The Supreme™ laryngeal mask airway (SLMA) is a single-use LMA with double lumen design that allows separation of the respiratory and the alimentary tract, hence potentially reducing the gastric volume and risk of aspiration. The purpose of this prospective cohort study is to evaluate the the role of the SLMA as an airway technique for women undergoing category 2 and 3 Cesarean delivery under general anesthesia.

**Methods:**

We recruited 584 parturients who underwent category 2 or 3 Cesarean delivery under general anesthesia, in which 193 parturients underwent category 2 and 391 parturients underwent category 3 Cesarean delivery. The primary outcome was insertion success rate at 1st attempt in SLMA insertion. The secondary outcomes included anaesthetic, obstetric outcomes and maternal side effects associated with airway device.

**Results:**

The 1st attempt insertion success rate was 98.3%, while the overall insertion success rate was 100%. The mean (Standard deviation) time to effective ventilation was 15.6 (4.4) seconds. Orogastric tube insertion was successful at the 1st attempt in all parturients. There was no clinical evidence of aspiration or regurgitation. No episodes of hypoxemia, laryngospasm or bronchospasm were observed intra-operatively. The incidence of complications was low and with good maternal satisfaction reported.

**Conclusions:**

The SLMA could be an alternative effective airway in category 2 and 3 parturients emergency Cesarean Delivery under general anesthesia in a carefully-selected obstetric population.

**Trial registration:**

Clinical Trials Registration: Clinicaltrials.gov Registration NCT02026882. Registered on December 31, 2013.

## Background

Pregnancy is associated with significant anatomical and physiological changes, associated with airway complications that contribute significantly to anesthesia-related maternal mortality [1, 2]. Although there is an increased risk of difficult airway and gastric aspiration in obstetrics, general anesthesia may still be necessary in emergent situations where Cesarean delivery is indicated for maternal or fetal reasons. The incidence of failed obstetric intubation could be as high as 1 in 224 [3–6].

With the introduction of the Classic™ laryngeal mask airway (LMA) in the 1980s, the use of LMAs in many airway management situations have been reported [7–9]. However, the use of LMA in obstetrics has been limited due to the risk of regurgitation and gastric aspiration. Reports have suggested the use of the Proseal™ laryngeal mask airway (PLMA) as an alternative airway in elective and emergency Cesarean deliveries [10–13]. The use of the PLMA in a large cohort of elective Cesarean deliveries suggested its role as an alternative to tracheal intubation in selected parturients [14]. The Supreme™ laryngeal mask airway (SLMA) is a single-use LMA with double lumen design to allow separation of the respiratory and the alimentary tract, reducing the risk of aspiration [15]. The use of the SLMA in 700 low risk parturients undergoing Cesarean delivery found a high 1st attempt insertion success rate of 98.0%, without airway complications [16]. In that study, 82.3% of the subjects were scheduled for elective Cesarean deliveries and 17.7% underwent urgent Cesarean deliveries. However, there is limited evidence of the use of SLMA in more emergent Cesarean deliveries.

This prospective study aims to evaluate the role of the SLMA as an airway technique for women undergoing category 2 and 3 Cesarean delivery under general anesthesia. Category 2 and 3 Cesarean deliveries refer to emergent situations where there are maternal or fetal compromise that are not immediately life-threatening, and those needing early delivery but no maternal or fetal compromise, respectively [17].

## Methods

This study was approved by the Institutional Review Board at the Quanzhou Women’s and Children’s Hospital in China. The Cesarean delivery rate at Quanzhou Women’s and Children’s Hospital is 35% and there are about 2000 women who have Cesarean delivery per year. The study was registered with the hospital ethics committee (dated 11th Nov 2013) and clinical trials registry (NCT02026882).

We recruited parturients who were healthy or with well-controlled medical conditions that underwent category 2 or 3 Cesarean delivery under general anesthesia at Quanzhou Women’s and Children’s Hospital, Fujian Province, China between December 2013 and November 2014. Parturients with body mass index (BMI) ≥ 35 kg/m^2^, potentially difficult airway (modified Mallampati grade 4, upper respiratory tract or neck pathology) or gastro-esophageal reflux disease (self-reported) were excluded from the study. All parturients were fasted for at least 4 h. The investigators provided information about the study to every parturient in the antenatal ward or delivery suite. If a subject had emergent indication for Cesarean section, the subject would be reconfirmed and recruited into the trial.

The majority of Cesarean deliveries at Quanzhou Women’s and Children’s Hospital are performed under general anesthesia using the SLMA, usually due to patient preference. The SLMA is already part of routine airway management in obstetrics in the institution. The size of SLMA used was based on manufacturer’s recommendations. However, at the discretion of the attending anesthesiologist, a more appropriate size could be selected based on parturient’s weight, BMI and mouth opening. Three investigators (Yao, Li, Yuan), each with more than 5 years of experience in the placement of SLMA for airway management in general anesthesia for Cesarean delivery, inserted the SLMA in this study.

Premedication with intravenous ranitidine was administered to parturients. Electrocardiogram, pulse oximetry, capnography and non-invasive blood pressure measurements were applied. After preoxygenation for 3 min, rapid sequence induction was carried out with cricoid pressure applied. Anesthesia was induced with propofol and succinylcholine intravenously. Fentanyl was administered perioperatively for intraoperative analgesia. Rocuronium was used for maintenance of muscle relaxation. Subsequently, the SLMA was inserted using the single-handed rotational technique recommended by the manufacturer, until resistance was met. The cuff was then inflated with air to a pressure of 60cmH_2_O, as measured by an intracuff pressure monitor. The volume of air needed to achieve this pressure was recorded. Upon SLMA placement, cricoid pressure was then released, the cuff inflated and the ability to ventilate confirmed.

Successful placement was confirmed by auscultation and the presence of end-tidal carbon dioxide on the capnogram. The number of attempts required to achieve successful placement, with an attempt defined as insertion and complete withdrawal of the device from the airway was recorded. The time to effective airway placement, defined as the interval from when the device was picked up until appearance of the 1st end-tidal carbon dioxide waveform, was also measured and recorded. After successful placement, a pre-mounted #14 orogastric tube was advanced through the gastric drainage aperture. Suction was performed at the beginning of the surgery and at the end before emergence. The successful orogastric tube insertion was confirmed by: a) aspiration of gastric contents; b) injection of air into orogastric tube via the large lumen whilst auscultating the stomach for a “swoosh” indicating gastric placement. The number of orogastric tube insertion attempts and failure to pass the orogastric tube were recorded. The oropharyngeal leak pressure was recorded by closing the adjustable pressure-limiting valve and insufflating the closed breathing system with 3 L/min of fresh gas flow. The peak airway pressure was recorded.

The investigators were allowed to use additional maneuvres (chin lift, jaw thrust, head extension) or reposition the SLMA if necessary to achieve airway patency. If successful placement could not be achieved (i) after 2 attempts, (ii) within 60 s, or (iii) before desaturation occurred (oxygen saturation < 92%), the airway would then be secured using direct laryngoscopy and endotracheal intubation. The surgical procedure was allowed to proceed if the following criteria were met: a square-wave capnograph tracing was present; the pilot cuff was inflated to 60 cmH_2_O and checked with a manometer; the bite block of the SLMA was sitting between the incisors; the gastric tube was inserted into the drain tube, and the position was checked using insufflation of 5 mL of air and auscultation over the epigastric region, followed by performing active/passive suction and then by passive drainage of the gastric tube and the leak pressure was checked, and the observed peak airway pressure achieved was ≥20 cmH_2_O.

Anesthesia was maintained with 1.5 to 2.0% sevoflurane and 50% nitrous oxide in oxygen. All parturients were placed in the left lateral tilt position using a wedge. During maintenance of anesthesia, complications including loss of airway, desaturation, inadequate ventilation and bleeding into the SLMA were recorded. The tidal volume was set from 6 to 10 mL/kg, and the respiratory rate ranged from 10 to 16 breaths/min to maintain an end-tidal carbon dioxide concentration of 30 to 40 mmHg. If there were signs of aspiration (perioperative hypoxemia, wheezing, crepitations upon lung auscultation, postoperative dyspnea), the parturient would be investigated with bronchoscopy or chest X-rays.

The obstetricians were given instructions (to reduce fundal pressure, or to use instrumental delivery such as forceps or vacuum extraction) to avoid excessive fundal pressure during fetal extraction. Upon surgery completion, muscle paralysis was reversed and the orogastric tube was suctioned and removed. The SLMA was removed and inspected for blood when regular spontaneous respiration returned, with the parturient conscious. Consciousness was defined as when patient was able to follow instructions to open eyes and mouth prior to removal of the LMA device. The incidence of sore throat and hoarseness were assessed by an independent assessor before discharge from the post anesthesia care unit.

Our primary outcome was insertion success rate at 1st attempt in SLMA insertion. Secondary anesthetic outcomes included: time to effective ventilation; oropharyngeal leak pressure; ventilation parameters (tidal volume, respiratory rate, peak airway pressure) to maintain effective oxygenation and ventilation, defined as the ability to maintain SpO_2_ ≥ 92% and an end-tidal carbon dioxide concentration of <50 mmHg, using inspired oxygen concentration ≤ 0.5 with respiratory rate 10 to 16 breaths/min and tidal volume 6 to 10 mL/kg; hemodynamic parameters (heart rate and blood pressure) for 6 min at induction. The amount and pH of gastric aspirate; incidence of regurgitation (clear or bile stained fluid seen during procedure or removal of airway device); incidence of aspiration (bile stained fluid seen in the lung during bronchoscopy or postoperative radiological evidence) and pH of the laryngeal surface of the SLMA. Obstetric outcomes included: neonatal weight; neonatal Apgar score at 1 and 5 min and umbilical cord venous pH. Maternal satisfaction with the anesthetic experience was assessed at 1 day after surgery.

### Statistical analysis

Primary outcome 1st attempt insertion success rate in SLMA insertion, secondary outcomes incidence of regurgitation, aspiration, blood stain on SLMA and hoarseness were treated as categorical data. All demographic, anesthetic and clinical categorical variables were summarized as frequency with corresponding proportion and continuous variables as mean (standard deviation (SD)) or median [interquartile range (IQR), min – max], whichever applicable. If the summary measure for continuous variable expressed in median (IQR, min - max) and mean (SD), then effect measure between categories of Cesarean delivery were defined as difference in medians with corresponding 95% confidence interval (CI) based on Hodges-Lehmann estimation method and mean difference (95% CI) respectively. However, effect measure for categorical variables was defined as risk difference with 95% CI. Association between categories of Cesarean delivery and categorical variables were compared using Fisher’s exact test while association between Cesarean delivery and continuous variables were compared using students’ t – test or Mann – Whitney U test, whichever applicable. Significance level was set at p - value <0.05 and all tests were two sided. Data analysis was generated using SAS software (SAS Institute Inc., Cary, NC, USA).

We performed a power analysis based on our previous experience estimate of 98% in 1st attempt insertion success rate of SLMA insertion [16]. An equivalence boundary of (93.5%, 99.3%) in 1st attempt insertion success rate in category 2 or 3 Cesarean delivery would be regarded as clinical equivalence. A sample size of 584 patients would achieve 89.7% power with the above mentioned equivalence limits, a one sided exact test with a significance level of 5% and retrospective estimate of 98% success rate.

## Results

We screened 619 parturients between December 2013 and November 2014 and 35 parturients did not give consent. There were 193 (33.0%) parturients who underwent category 2 Cesarean delivery and 391 (67.0%) parturients underwent category 3 Cesarean delivery. There was no withdrawal or dropout.

Demographic and clinical characteristics of the parturients are summarized in Table 1. The mean (SD) duration of surgery was 29.5 (9.4) minutes with 221 (37.8%) parturients who were in labour. Women in the category 2 had lower mean (SD) BMIs (26.4 (3.59) kg/m^2^), lower mean (SD) gestational age (36.9 (2.52) weeks) compared with the category 3 (27.4 (3.86) kg/m^2^), 38.5 (1.19) weeks, respectively). As expected, there was a higher percentage of parturients in category 2 Cesarean delivery who were in labor compared to category 3 Cesarean delivery. All parturients were fasted for at least 4 h.

**Table 1:** Demographic and clinical outcomes. Values are presented as frequency (%), mean (SD) and median [IQR, min - max]

Characteristic	All Parturients *n* = 584	Category 2 Parturients *n* = 193	Category 3 Parturients *n* = 391	Effect measure (95% CI)	*P* -values
Age (years), mean (SD)^a^	28.9 (4.14)	28.5 (4.15)	29.1 (4.13)	0.58 (−0.13, 1.30)	0.1092
BMI (kg /m^2^), mean (SD)^a^	27.1 (3.80)	26.4 (3.59)	27.4 (3.86)	0.95 (0.31, 1.58)	0.0037
Gestational age (weeks), mean (SD)^a^	37.9 (1.90)	36.9 (2.52)	38.5 (1.19)	1.6 (1.23, 1.98)	<0.0001
Active labor, n(%)^c^	221 (37.8)	169 (87.6)	52 (13.3)	−0.74 (−0.68, −0.80)	<0.0001
ASA, median [IQR, min - max]^b^	2 [2–2, 1–3]	2 [2–2, 1–3]	2 [2–2, 1–3]	–	0.0787
Mallampati score, median [IQR, min - max]^b^	2 [1–2, 1–4]	2 [1–2, 1–3]	2 [1–2, 1–4]	–	0.8871
Duration of surgery (minute), mean (SD)^a^	29.5 (9.4)	30.4 (10.8)	29.0 (8.6)	−1.38 (−3.14, 0.38)	0.1233

Note: Effect measure is expressed as ^a^mean difference with 95% CI, ^b^difference in median with 95% CI or ^c^risk difference with 95% CI. Body Mass Index = BMI; American Society of Anesthesiologists = ASA

Anesthetic outcomes are presented in Table 2. The SLMA provided an effective airway in all parturients (overall insertion success rate of 100%), 574 (98.3%) at the 1st insertion attempt and 10 (1.7%) at the 2nd insertion attempt. The average (SD) time to reach effective ventilation was 15.6 (4.4) seconds. No additional maneuvers were needed during the insertion attempts. There was no clinical evidence of aspiration or regurgitation in any parturients. The median [IQR, min, max] difference between seal pressure and peak airway pressure was 9 [5–13, −8 - 24] cmH_2_O. The peak airway pressure and seal pressure were lower in category 2 (17 [14–19] cmH_2_O, 27 [24–29] cmH_2_O) compared with category 3 (18 [16–22] cmH_2_O, 28 [25–30] cmH_2_O). There were 6 (3.1%) parturients in the category 2 and 7 (1.8%) parturients in the category 3 Cesarean delivery with negative seal pressure and peak airway pressure difference. The peak airway pressure was transient (less than 1 min) in these cases and there was no airway complication.

**Table 2:** Anaesthetic outcomes. Values are presented as frequency (%), mean (SD) and median [IQR, min - max]

Characteristic	All Parturients *n* = 584	Category 2 Parturients *n* = 193	Category 3 Parturients *n* = 391	Effect measure (95% CI)	*P* - value
Airway insertion attempt, *n*(%)^c^				−0.01 (0.08, −0.09)	0.7367
1st attempt insertion success	574 (98.3)	189 (97.9)	385 (98.5)		
2nd attempt insertion success	10 (1.7)	4 (2.1)	6 (1.5)		
Time to effective ventilation (second), mean (SD)^a^	15.6 (4.4)	15.3 (4.5)	15.7 (4.4)	0.48 (−0.29, 1.25)	0.2230
Peak airway pressure (cm H_2_O), median [IQR, min - max]^b^	18 [15–21, 11–32]	17 [14–19, 11–27]	18 [16–22, 11–32]	1.00 (0.26, 1.74)	<0.0001
Seal pressure (cm H_2_O), median [IQR, min - max]^b^	27 [25–30, 17–39]	27 [24–29, 18–36]	28 [25–30, 17–39]	1.00 (0.36, 1.64)	0.0003
Seal – Peak airway pressure (cm H_2_O), median [IQR, min - max]^b^	9 [5–13, −8 - 24]	9 [6–13, −8 - 13]	9 [5–13, −3 - 24]	0.00 (−1.11, 1.11)	0.3594
Cuff volume (mL), mean (SD)^a^	25.0 (2.9)	25.0 (2.6)	25.0 (3.0)	0.03 (−0.44, 0.5)	0.9073
Number of attempts at orogastric tube insertion, median [IQR, min - max]^b^	1 [1–1, 1–1]	1 [1–1, 1–1]	1 [1–1, 1–1]	–	1.0000
Volume of gastric aspirate (mL), mean (SD)^a^	14.2 (14.3)	12.1 (7.4)	15.2 (16.6)	3.16 (1.21, 5.11)	0.0016
pH of gastric aspirate, mean (SD)^a^	2.3 (0.8)	2.4 (0.9)	2.3 (0.8)	−0.08 (−0.24, 0.07)	0.2845
Respiratory rate (breaths /minute), median [IQR, min - max]^b^	12 [12–12, 10–17]	12 [12–12, 10–15]	12 [12–12, 10–17]	–	0.5250
Tidal volume (mL), median [IQR, min - max]^b^	480 [450–500, 360–650]	470 [450–500, 360–600]	480 [450–500, 380–650]	10.00 (−2.48, 22.48)	0.0002
pH on laryngeal surface, mean (SD)^a^	7.0 (0.5)	7.0 (0.6)	7.1 (0.4)	0.04 (−0.05, 0.13)	0.3985

Note: Effect measure is expressed as ^a^mean difference with 95% CI, ^b^difference in median with 95% CI or ^c^risk difference with 95% CI

The mean (SD) volume of air to maintain an effective airway was 25.0 (2.9) mL. Orogastric tube insertion was successful at 1st attempt in all parturients. The volume of gastric aspirate (12.1 (7.4) mL) was lower in category 2 compared with category 3 (15.2 (16.6) mL). No episodes of hypoxemia, laryngospasm or bronchospasm were observed intra-operatively.

The incidence of airway complications was low in this study. Eight (1.4%) parturients had blood on SLMA postoperatively. Thirty-eight (6.4%) and 4 (0.7%) parturients reported sore throat and hoarseness respectively. The presence of blood on SLMA was seen more parturients in category 2 (7 (3.6%)) than in category 3 (1 (0.3%)). Overall, the maternal satisfaction was high with the mean (SD) of 86.1 (8.6) on a scale of 0–100 during delivery. However, the parturients in category 2 had lower maternal satisfaction (84.6 (9.2)) compared with category 3 (86.9 (8.2)). Obstetric outcomes and maternal side effects associated with airway device are summarized in Table 3.

**Table 3:** Obstetric outcomes and maternal side effects associated with airway device. Values are presented as frequency (%), mean (SD) and median [IQR, min-max]

Characteristic	All Parturients *n* = 584	Category 2 Parturients*n* = 193	Category 3 Parturients *n* = 391	Effect measure (95%CI)	*P* - value
Neonatal weight (kg), mean (SD)^a^	3.0 (0.5)	2.7 (0.5)	3.2 (0.4)	0.51 (0.42, 0.60)	<0.0001
Neonatal APGAR 1 min, median [IQR, min-max]^b^	9 [9–10, 3–10]	9 [8–10, 3–10]	10 [9–10, 7–10]	–	<0.0001
Neonatal APGAR 5 min, median [IQR, min-max]^b^	10 [10–10, 5–10]	10 [9–10, 5–10]	10 [10–10, 8–10]	–	<0.0001
Neonatal venous cord pH, mean (SD)^a^	7.3 (0.1)	7.3 (0.1)	7.3 (0.1)	0.003 (−0.01, 0.01)	0.5495
Presence of blood on SLMA, *n* (%)^c^	8 (1.4)	7 (3.6)	1 (0.3)	−0.03 (0.05, −0.12)	0.0023
Sore throat, *n* (%)^c^	38 (6.5)	14 (7.3)	24 (6.1)	−0.01 (0.08, −0.10)	0.5970
Hoarseness, *n* (%)^c^	4 (0.7)	0 (0.0)	4 (1.0)	0.01 (0.10, −0.08)	0.3077
Maternal satisfaction, mean (SD)^a^	86.1 (8.6)	84.6 (9.2)	86.9 (8.2)	2.29 (0.76, 3.83)	0.0034

Note: Effect measure is expressed as ^a^mean difference with 95% CI, ^b^difference in median with 95% CI or ^c^risk difference with 95% CI

## Discussion

This study demonstrated the use of the SLMA in providing adequate ventilation and oxygenation for 584 parturients receiving general anesthesia for more emergent category 2 and 3 Cesarean delivery, which there has not been any large prospective cohort study done before. The SLMA 1st attempt insertion success rate was 98.3% with no airway complications and the overall success rate was 100%. There was low incidence of airway complications, with good maternal satisfaction.

The high 1st attempt insertion success rate of 98.3% could be attributed to: insertion of SLMA performed using technique recommended by manufacturers, insertion by experienced anesthesiologists and the routine use of SLMA for general anesthesia in Cesarean delivery at the study site. This high success is comparable to rates reported by similar LMA studies which range from 97.7 to 98.0% using the LMA for airway management for Cesarean delivery [14, 16, 18]. This study with more emergent indications for Cesarean delivery contributes to the growing literature that supraglottic airway devices could be used for airway management by experienced anesthesiologists during Cesarean delivery.

The obstetric population is considered to be at risk for regurgitation and aspiration, due to increased intragastric pressure and lower esophageal sphincter pressure. The risk is further increased if there is concomitant obesity, being in labour or receiving opioid analgesia [19, 20]. Our study parturients would at risk of regurgitation and gastric aspiration [21]. However, we did not detect any clinical evidence of regurgitation or aspiration. The ability of the double lumen system of 2nd generation LMA (SLMA, PLMA) could attenuate and prevent gastric fluid at the hypopharynx from entering the airway [22–26].

Several studies have evaluated the role of supraglottic airway devices in parturients receiving general anesthesia for Cesarean delivery. The prospective study of 1067 parturients scheduled for elective Cesarean delivery by Han et al. in 2001 demonstrated a 1st attempt insertion rate of 99.0%, using the Classic™ LMA, with no aspiration or regurgitation detected [18]. The use of PLMA in 3000 parturients by Halaseh et al. demonstrated a high 1st attempt insertion rate of 99.7%. Although 1 parturient had gastric content regurgitation after insertion, there was no clinical evidence of aspiration [14]. More recently, our group demonstrated the use of SLMA in 700 parturients with 1st attempt insertion rate of 98.0% and no clinical evidence of regurgitation or aspiration [16]. Of note, these studies selected parturients who were scheduled for elective and urgent Cesarean delivery and not in active labour.

This study attempts to investigate parturients with more emergent obstetric indications and also 38.4% of the parturients were in active labour. Although there were demographic and clinical outcome differences between category 2 and category 3 in our study, the anesthetic outcomes and side effects associated with airway device were similar, indicating SLMA could be still considered for airway management in either group.

The incidence of sore throat in our study was 6%, which was higher comparing to other studies using LMA Classic or LMA ProSeal [14, 18]. However, it was comparable with our previous study [16]. It is interesting that peak airway pressure and peak airway pressure were statistically different between the category 2 and category 3 groups. However, the clinical relevance is unclear. Another noteworthy phenomenon is that insertion success was very high despite the cricoid pressure being maintained. Some anesthetists advocate release of cricoid pressure on insertion of an LMA, to allow the tip to enter the postcricoid hypoparyngeal space. This might be a feature of the relative rigidity of the SLMA compared to other devices which are softer in the tip such as the PLMA.

There are several limitations in this study. We carefully selected the parturients to reduce the risk of gastric regurgitation or aspiration and all parturients were fasted for at least 4 h. Thus, results from this study should not be extended to obstetric populations deemed to be at high risk of regurgitation or aspiration. Furthermore, the study population was fairly homogeneous in respect to BMI and phenotype, the results may be different from populations with very varied ethnic backgrounds or where selection of the patients for LMA use is less stringent. Although there was high 1st attempt insertion rate, gastric regurgitation and aspiration would be of even greater interest to clinicians. Given that aspiration risk in high risk parturients could be as high as 1 in 667 [27], we would expect the aspiration risk in our study to be lower. Our study is underpowered to detect these rare events. As this study was conducted in a centre with routine high use of SLMA in general anesthesia for Cesarean delivery, the findings may not be applicable in centres where the use of supraglottic devices is less common.

The recent Difficult Airway Society- Obstetric Anaesthesia Association has recommended the use of second generation Supraglottic airway devices (SAD), but does not specifically state the particular SAD to be used. Although the insertion success rate is high and insertion time short, tracheal intubation through the LMA Supreme in any case of difficulty is not practical. The results of this study cannot be extrapolated to patients with difficult airway as most use of LMA devices are in emergency failed intubation situations.

## Conclusions

In conclusion, our study demonstrated the effective use of the SLMA as an alternative airway device in providing ventilation and oxygenation in parturients receiving general anesthesia for category 2 and 3 emergency Cesarean delivery in a carefully selected obstetric population. There was no clinical evidence of gastric regurgitation or aspiration, and side effects were minimal. While rapid sequence intubation with tracheal intubation remains the 1st line airway management in general anesthesia for Cesarean delivery, the SLMA may be considered as a useful alternative airway. These findings could provide further support on the use of supraglottic airway devices especially during emergency difficult obstetric airway situations using current guidelines [28]. Up to date, there is only one randomized controlled trials comparing LMA ProSeal with endotracheal tube for elective Cesarean sections that has been performed in only 60 parturients [29]. Future randomized controlled trials with adequate sample size are needed to compare the use of second generation of SAD in emergent obstetric patients.

## Abbreviations

ASA: American Society of Anaesthesiologists
BMI: Body mass index
CI: Confidence interval
IQR: Interquartile range
LMA: Classic™ laryngeal mask airway
max: maximum
min: minimum
PLMA: Proseal™ laryngeal mask airway
SAD: Supraglottic airway devices
SD: Standard deviation
SLMA: Supreme™ laryngeal mask airway
